# Change of strategy is required for malaria elimination: a case study in Purworejo District, Central Java Province, Indonesia

**DOI:** 10.1186/s12936-015-0828-7

**Published:** 2015-08-16

**Authors:** E Elsa Herdiana Murhandarwati, Anis Fuad, Sulistyawati, Mahardika Agus Wijayanti, Michael Badi Bia, Barandi Sapta Widartono, Kuswantoro, Neil F Lobo, Supargiyono, William A Hawley

**Affiliations:** Center for Tropical Medicine and Department of Parasitology, Faculty of Medicine, Universitas Gadjah Mada, Yogyakarta, Indonesia; Department of Public Health, Faculty of Medicine, Universitas Gadjah Mada, Yogyakarta, Indonesia; Department of Public Health, Universitas Ahmad Dahlan, Yogyakarta, Indonesia; Politeknik Kesehatan, Kementrian Kesehatan Kupang, Kupang, Indonesia; Cartography and Remote Sensing Department, Faculty of Geography, Universitas Gadjah Mada, Yogyakarta, Indonesia; District Health Office of Purworejo, Purworejo, Indonesia; Eck Institute for Global Health, University of Notre Dame, Notre Dame, IN 46556 USA; UNICEF, Jakarta, Indonesia

**Keywords:** Malaria elimination, Health system, Decentralization policy, Strategies, Purworejo

## Abstract

**Background:**

Malaria has been targeted for elimination from Indonesia by 2030, with varying timelines for specific geographical areas based on disease endemicity. The regional deadline for malaria elimination for Java island, given the steady decrease of malaria cases, was the end of 2015. Purworejo District, a malaria-endemic area in Java with an annual parasite incidence (API) of 0.05 per 1,000 population in 2009, aims to enter this elimination stage. This study documents factors that affect incidence and spatial distribution of malaria in Purworejo, such as geomorphology, topography, health system issues, and identifies potential constraints and challenges to achieve the elimination stage, such as inter-districts coordination, decentralization policy and allocation of financial resources for the programme.

**Methods:**

Historical malaria data from 2007 to 2011 were collected through secondary data, in-depth interviews and focus group discussions during study year (2010–2011). Malaria cases were mapped using the village-centroid shape file to visualize its distribution with geomorphologic characteristics overlay and spatial distribution of malaria. API in each village in Purworejo and its surrounding districts from 2007 to 2011 was stratified into high, middle or low case incidence to show the spatiotemporal mapping pattern.

**Results:**

The spatiotemporal pattern of malaria cases in Purworejo and the adjacent districts demonstrate repeated concentrated occurrences of malaria in specific areas from 2007 to 2011. District health system issues, i.e., suboptimal coordination between primary care and referral systems, suboptimal inter-district collaboration for malaria surveillance, decentralization policy and the lack of resources, especially district budget allocations for the malaria programme, were major constraints for programme sustainability.

**Conclusions:**

A new malaria elimination approach that fits the local disease transmission, intervention and political system is required. These changes include timely measurements of malaria transmission, revision of the decentralized government system and optimizing the use of the district capitation fund followed by an effective technical implementation of the intervention strategy.

## Background

The WHO Global Malaria Eradication Programme collapsed in the 1970s, resulting in an increase of malaria in many regions which had previously experienced significant control [1]. However, during the past decade scale-up of interventions against malaria, including insecticides-treated nets (ITNs), indoor residual spray (IRS), rapid diagnostics testing (RDT), and artemesinin-based combination therapy (ACT), has led to significant declines in malaria globally. The Bill and Melinda Gates Foundation (BMGF) [with endorsement by the WHO and Roll-Back Malaria (RBM) Partnership] announced global eradication of malaria as a Foundation priority in 2007. This goal is supported by large malaria control support efforts, such as the Global Fund to Fight AIDS, Tuberculosis and Malaria, and the US President’s Malaria Initiative. A parallel research agenda has been developed to facilitate discovery, development and implementation of novel interventions to facilitate malaria elimination.

Malaria was first formally reported in Indonesia in 1854. Almost the entire population of Indonesia was at risk before the National Malaria Control Programme was founded in 1950 [2]. Since Indonesia’s independence in 1945, malaria control has been intensively conducted through the Malaria Control Programme (1945–1958) [3, 4] and the Malaria Eradication Programme (1959–1968) which focused on DDT spraying and treatment of fever cases with chloroquine. This was followed by the Malaria Control Phase (1969–1999) and Indonesia Roll Back Malaria Campaign (2000 to present) which focuses on malaria case detection and surveillance and integrated activities as recommended by WHO [5].

Although there have been fluctuations in global malaria support efforts, thanks to strong national commitment, some countries are successful in their progress toward malaria elimination, including Indonesia. Before any control was implemented in Indonesia, it is estimated that there were 30 million cases and 120,000 deaths due to malaria annually in 1919 [6]. Although the decline in malaria was not always steady due to political turmoil in 1966 and sudden implementation of decentralization policy in 2000, an overall temporal decline has been observed. In 2011, the total number of suspected malaria cases was 2.4 million out of 240 million population, with the total confirmed malaria cases was 475,508, split nearly evenly between *Plasmodium falciparum* (47%), followed by *Plasmodium vivax* (45%) [7]. However, this success is not distributed evenly throughout Indonesia, with the eastern part of the country remaining malaria-endemic and the large islands of Sulawesi, Kalimantan and Sumatra having higher incidence than Java [8, 9]. Therefore, Indonesia, along with India and Myanmar contributed to 95% of reported malaria cases and deaths in Southeast Asia in 2011 [10]. The Indonesian National Malaria Programme aspires to eliminate malaria from the country by 2030 [11]. During 2004–2009, the annual parasite incidence (API) in Bali and Java was approximately 0.15–0.17 per 1,000, which was below the target (0.25 per 1,000) [12]. Thus, if this trend continues, malaria elimination is expected in Java and Bali by 2015.

The transmission dynamics of malaria are determined by the local environment, vector species bionomics, human behaviour, and parasite biology. The island of Java is the centre of economic growth in Indonesia as evidenced by a high Gross Domestic Product relative to outer islands [13]. Furthermore, 70% of the Indonesian population resides in Java. Population mobility-related issues such as transmigration (due to cultural activity, jobs, etc.) to other islands (particularly to islands in malaria-endemic areas) and urbanization are important factors that contribute significantly to malaria transmission in Java.

Purworejo is a district in the Province of Central Java. Purworejo is a part of the Menoreh Hills and is endemic for malaria transmission [14]. Its bordering districts (Kebumen, Wonosobo, Magelang, and Kulon Progo) are also endemic for malaria. During1986-1995, API in Purworejo was reduced around 2–11 per 1,000 [14]. However, a drastic surge occurred in 2000, when the API in Purworejo reached 44.62 per 1,000 [15]. The launching of *Gebrak Malaria* (Actions to Re-Eliminate Malaria) by the Indonesian Ministry of Health in 2000 successfully reduced the API to 0.77 per 1,000 in 2004.

**Fig. 1:**
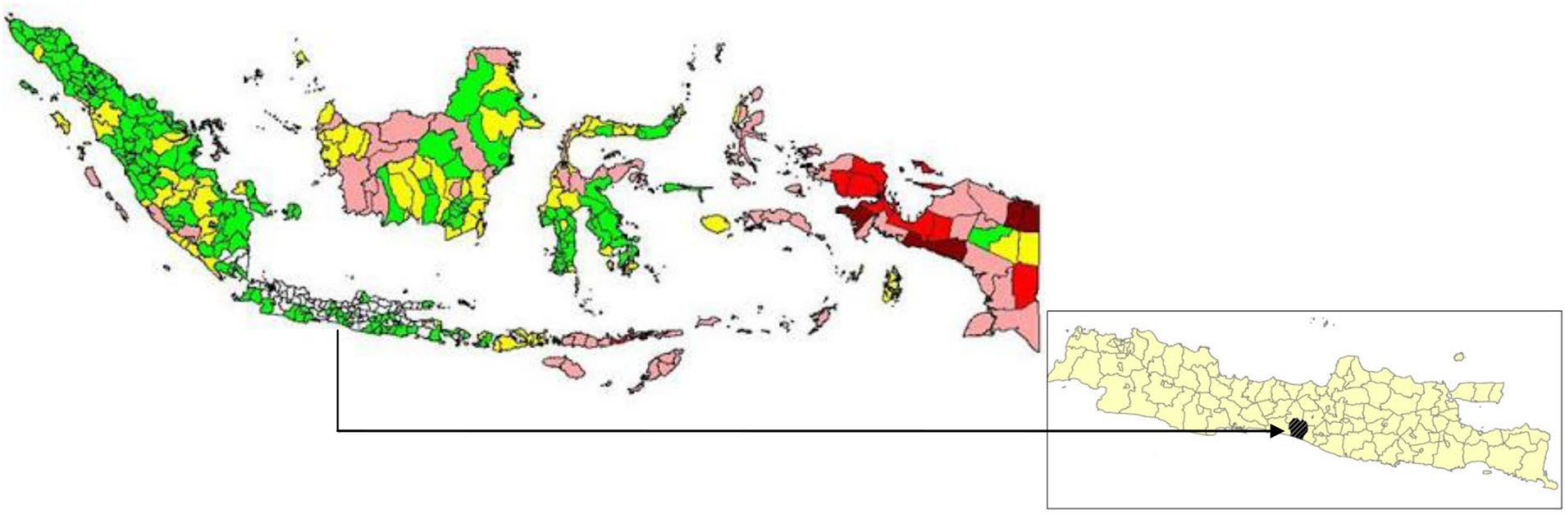
Malaria endemicity map of Indonesia in 2010. Annual parasite incidence in each district was stratified as malaria free (*white*), low case incidence/LCI (API <1%, *green*), middle case incidence/MCI (API 1–5 per 1,000 population, *yellow*), high case incidence/HCI I (API 5–49 per 1,000 population, *pink*), high case incidence/HCI II(API 50–100 per 1,000 population, *red*), and high case incidence III/HCI III (API >100%, *dark red*) (*Source DitJen P2PL RI, 2010*).* Inset* is Java island, the area in *black* is the study area, Purworejo.

At national level, malaria cases have decreased over the last 5 years. The API was 2.89, 2.47, 1.85, and 1.96 per 1,000 population in 2007, 2008, 2009, and 2010, respectively. In 2010, it was estimated that 117,351,457 people in Indonesia were at risk of malaria [16]. Although API was relatively low at the national level, there was a large difference between the API within and outside Java island (Fig. 1). The API values in provinces outside Java varied between 0.24 and 18.03 per 1,000 population, whereas in Java the highest API was 0.43 per 1,000 in West Java Province. In Central Java Province, API was 0.12, 0.07, 0.08 and 0.10 per 1,000 in 2007, 2008, 2009, and 2010, respectively (Table 1) with the number of people at risk of 21,430,044. The contribution of malaria cases in Purworejo towards the total number of malaria cases at Central Java Province, Java island and national level in 2010 is depicted at Table 2.

**Table 1:** API at national level, Java–Bali island, Central Java Province and Purworejo District during from 2007 to 2010

	API (‰)				
	2007	2008	2009	2010	2011
National	2.89	2.47	1.85	1.96	1.75
Central Java	0.06	0.05	0.05	0.10	0.11
Purworejo	0.57	0.61	0.47	0.49	1.34

*Sources* Dit.Jen. PP & PL, Kemenkes RI, 2013; Central Java Health Profile, 2009 and Central Java Health Profile 2012.

**Table 2:** The contribution of malaria cases in Purworejo District in 2010 to the total number of its cases at Province, Java and National levels in the same year

	Population at risk	Number of malaria confirmed cases	Contribution of malaria cases in Purworejo to each level (%)	API (‰)
Indonesia	117,351,457	229,819	0.16	1.96
Java	36,576,341	3,370	11.0	0.67
Central Java	21,430,044	2,098	17.7	0.10
Purworejo	766,328	372	100	0.49

Adopted from Dit.Jen. PP & PL, Kemenkes RI, 2011.

In 2010 (the year of the commencement of this study) the API in Purworejo was 0.49 per 1,000 and this district was categorized as a low case incidence (LCI) site, indicating that malaria cases are below one per 1,000 population annually. Comprehensive assessment of micro-epidemiology [17] is critical to evaluate preparedness of Purworejo in entering the malaria elimination phase. This study aims to evaluate factors that affect the incidence and spatial distribution of malaria in Purworejo and its adjacent districts, i.e., geomorphology, topography, health system, and decentralization policy, including financial resources. To achieve this goal, historical data of malaria and spatial distribution of malaria from 2007 to 2011 were collected through secondary data, in-depth interviews and focus group discussions during the study year (2010–2011). Elimination efforts, challenges, constraints, and lessons learned from Purworejo and its surrounding district areas are valuable for public health policy planning on malaria in other endemic areas, either inside or outside Indonesia, with similar characteristics.

## Methods

### Study sites

The district of Purworejo stretches between 109°47′28″ and 110°8′20″ East and between 7°32″ and 7°54″ South. It has an area of 1,034.82 sq km, consisting of a hilly mountainous region in the north, more level rice-growing areas in the middle, and lower elevations by the southern coastal area. Elevation varies from 0 to 420 m above sea level. This region has a wet tropical climate with mean daily temperature ranging from 19 to 28°C and humidity around 70–90%. Rainfall is highest in rainy season (~450 mm) and could reach 0 mm in dry season [18]. The southern part of Purworejo is bordered by the Indian Ocean. The district consists of 16 sub-districts (*kecamatan*) and 494 villages. In 2010, when the study was initiated, Purworejo had a population of 898,631 (*Source: District Health Office of Purworejo, 2010*).

### Malaria documentation

Malaria cases from Purworejo and the neighbouring districts (Kebumen, Wonosobo, Magelang, and Kulon Progo) from 2007 to 2011 were collected from all District Health Offices (DHOs). API in each village was stratified using the village as the smallest unit and was mapped spatiotemporally.

Historical data related to health care systems, malaria diagnosis, malaria control activities, ACT, and data regarding cross-border and cross-sector activities, including the malaria control local budget during 2007–2011, were collected through in-depth interviews, focus group discussions and observation during the study period (November 2011–October 2012). In-depth interviews were conducted with the malaria programme manager and the staff of the Purworejo DHO, heads of primary health centres (PHCs), malaria programme managers at PHC level, and from clinicians at the referral hospital and Wonosobo DHO. Focus group discussions (FGDs) were conducted six times and included 38 informants, i.e., village malaria workers (VMWs), midwives, paramedics, and community leaders. These informants included those that work under the PHC, and those that have retired but still serve the community in an unofficial capacity. Each FGD sessions was conducted for 60 min. The in-depth interviews also included 15 informants: the head of PHCs, general practitioners working in PHCs, malaria specialists working at the referral hospital, district pharmacist, district health officer working on either malaria or infectious diseases control and the Head of DHOs of the surrounding districts. FGDs and in-depth interviews were recorded, transcribed, interpreted, and discussed with the informants and the findings were then confirmed with the DHOs.

### Ethical considerations

This study was reviewed and approved by Institutional Review Boards for the ethical conduct of research on human subjects at the Universitas Gadjah Mada, Yogyakarta, Indonesia. REF: KE/FK/760/EC. Informed consent was collected from informants (for interviews and FGDs) before their participation in this study.

## Results

### The topography of Purworejo and neighbouring districts

Purworejo has a varied topography consisting of mountains, hills and plains. This geomorphology is shared with the neighbouring areas in the north, i.e., Wonosobo, Magelang and Kulon Progo districts. The northern region of Purworejo (which is also part of South Serayu Hills) consists of Halang and Peniron formations dominated by sedimentary rocks. Erosion from those areas results in transfer of soil to the urban plains.

**Fig. 2:**
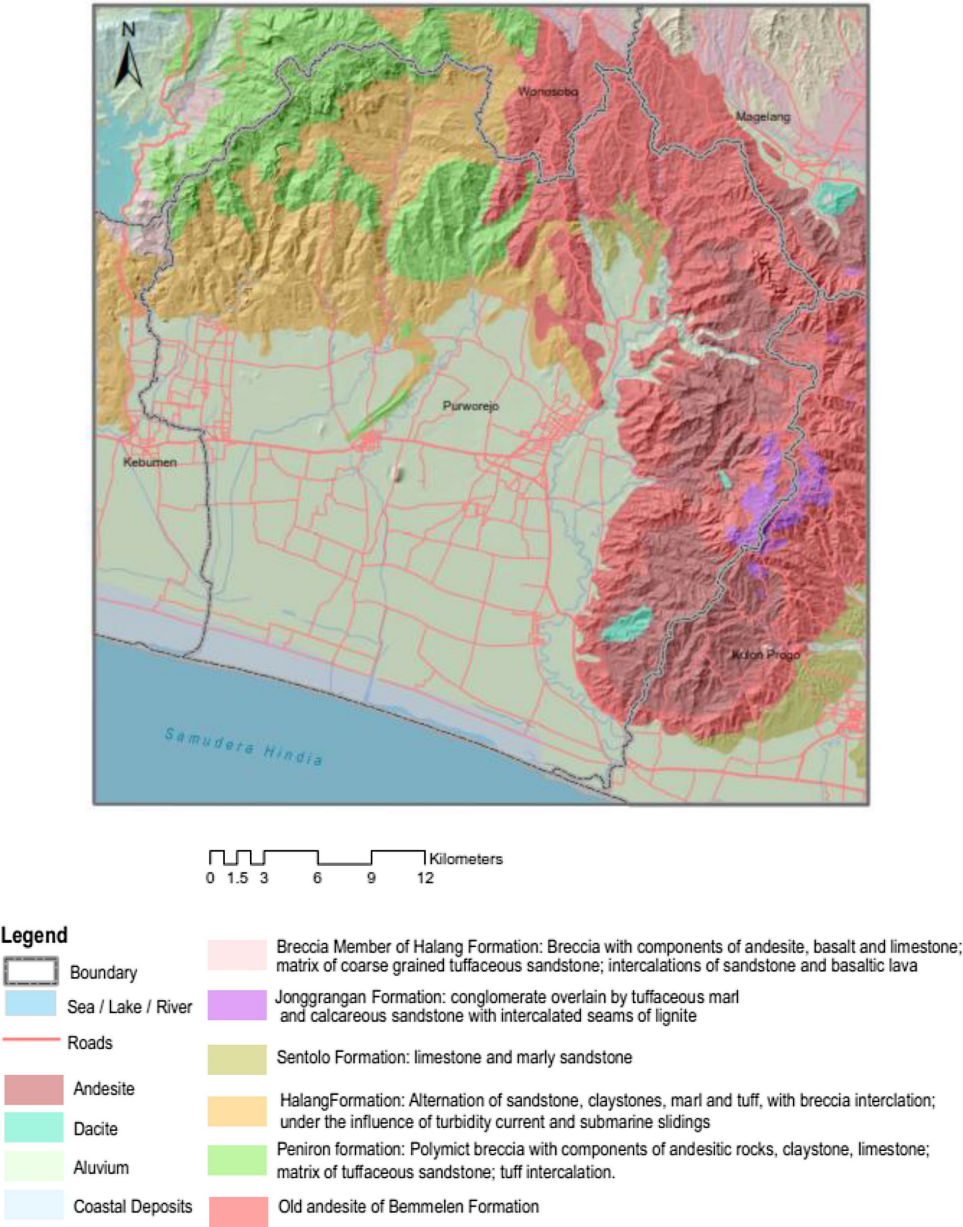
Topography and lithography of Purworejo.

The northeast, east and southeast area of Purworejo share similar geomorphology with Magelang and Kulon Progo districts, known as the Menoreh Hills, dominated by andesite, old andesite and Bemmelen formation (Fig. 2). As a consequence of its rough topography and andesite rock type, these areas have a low porosity that allows the accumulation of stagnant water on rocky outcrops. This occurs in the northern area as well, although the relief is not as rough as the eastern part. This condition supports the occurrence of a high water table (*cerukan air*) which is conducive for malaria vector breeding sites in this region, which includes ***Anopheles sundiacus, *Anopheles barbirostris*, *Anopheles annularis*, *Anopheles minimus*, *Anopheles kochi*, *Anopheles aconitus*, *Anopheles tessellatus*, *Anopheles vagus*, *Anopheles subpictus*, *Anopheles indefinitus*, *Anopheles maculatus*, *Anopheles flavirostris*, *Anopheles balabacensis*, and *Anopheles barbumbrosus* [19]. Vector species known to carry sporozoites as detected by PCR and ELISA methods include: *A*. *aconitus*, *An. maculatus*, *An. balabacensis*, *An. vagus* and *An. barbirostris*. Both *P. vivax* and *P. falciparum* were found. The confirmation of some *Anopheles* as malaria vectors in Purworejo or its adjacent districts has been reported by others [20, 21] as well as vector density studies and seasonal correlations [15, 22, 23]. The vector species found here bite primarily outdoors throughout the night and although primarily zoophilic, some species (*An. aconitus* and *An. balabacensis*) were found to be more anthropophilic (St Laurent B, pers comm).

### Spatial distribution of malaria in Purworejo and surrounding districts (2007–2011)

**Fig. 3:**
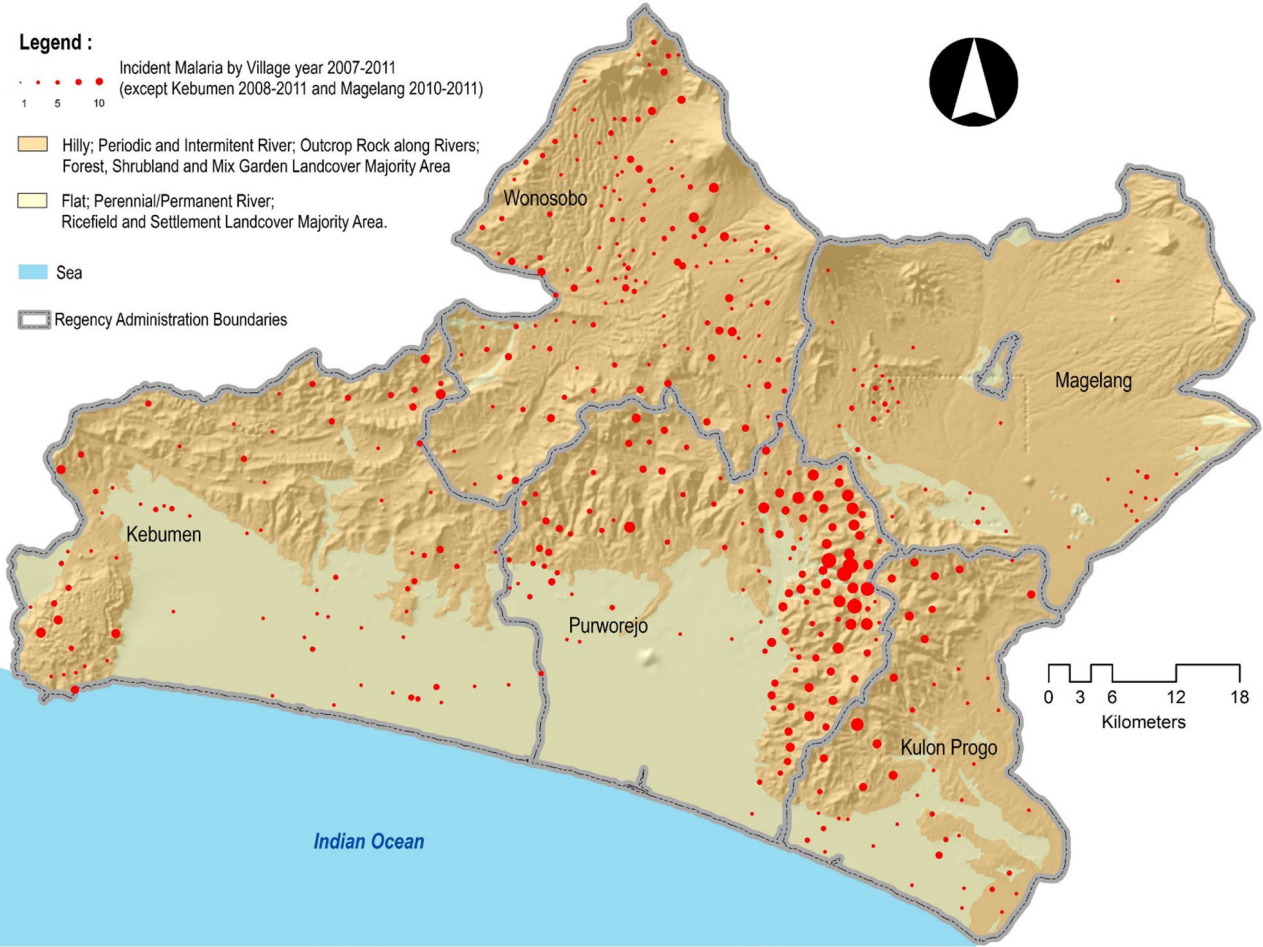
Malaria cases from Purworejo and adjacent districts. Malaria cases were mapped using the village-centroid shape file to visualize its distribution with geomorphologic characteristics overlay.

Malaria cases from Purworejo and the adjacent districts were mapped by village with a geomorphologic overlay (Fig. 3). This map shows that malaria cases were mostly located in hilly areas, inter-district borders and closer to periodic or intermittent rivers than those within flat, rice-growing areas.

**Fig. 4:**
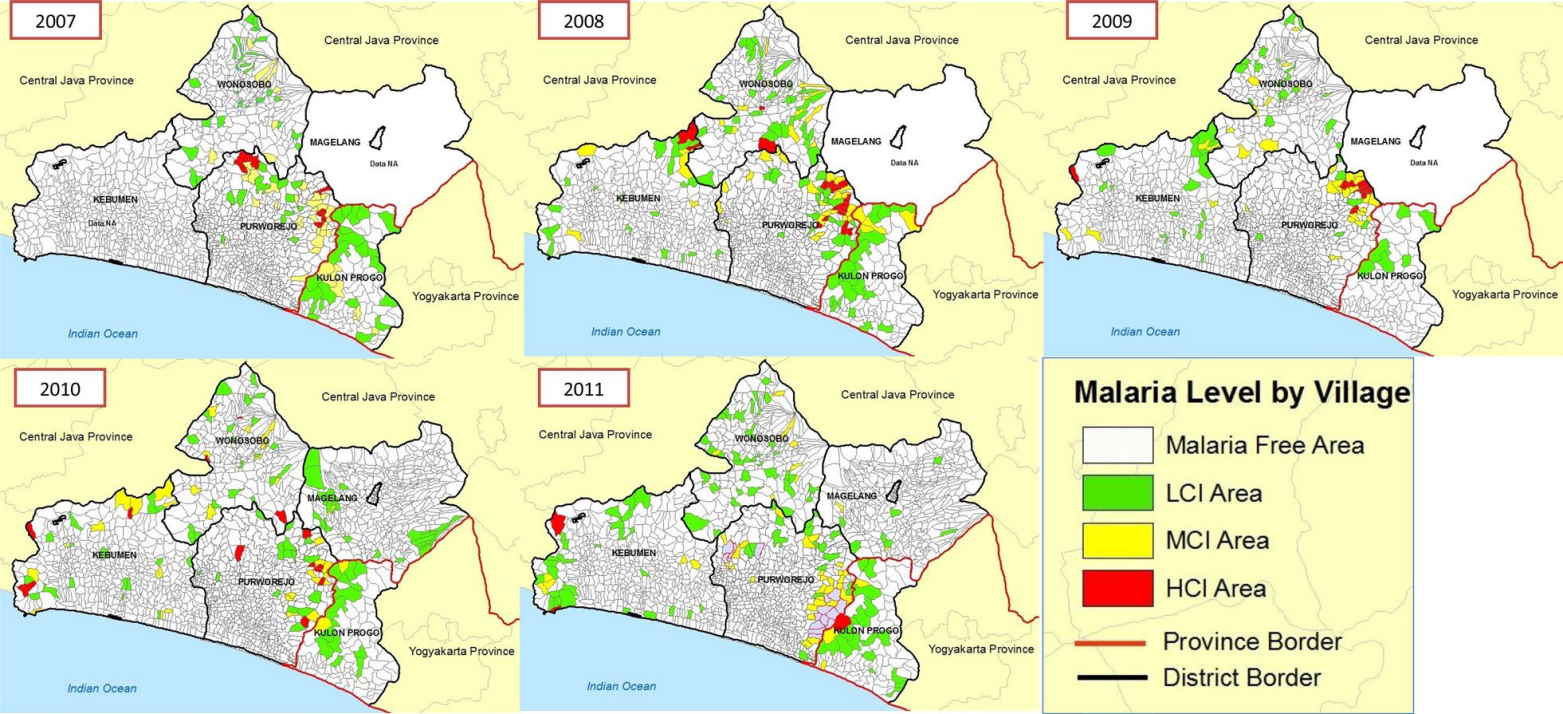
Spatio-temporal mapping of malaria in Purworejo and its surroundings districts (2007–2011). Malaria incidence in each village was stratified as high case incidence/HCI (*red*), middle case incidence/MCI (*yellow*) and low case incidence/LCI (*green*).

API in each village in Purworejo and its surroundings districts was temporally (2007–2011) stratified by incidence (Fig. 4). In 2007, it was shown that middle case incidence (MCI) villages were more dominant than LCI or high case incidence (HCI) villages and the distribution of HCI villages was located in the north and northeast areas. In 2008, the number of villages with malaria increased but were more concentrated in northeast and eastern areas at the border with Magelang and Kulon Progo. Although there was a decrease in the number of HCI villages in 2009 and 2010, it was followed by a sharp increase both in the total number of villages with malaria and HCI villages in 2011, moving from east to south at the border of Kulon Progo. A similar situation occurred in Kulon Progo, particularly in 2009 when the number of malaria-endemic villages decreased but then increased sharply in 2010 with one HCI village in 2011 [24].

Malaria cases in Kebumen district seem to bear no relation to those in Purworejo district. The spatio-temporal pattern of malaria in Purworejo and its surrounding districts supports the fact that malaria has existed in areas that share similar geomorphology regardless of administrative boundaries. Given that malaria cases often and repeatedly occur in hilly areas but not always in the same villages, the focus of interventions should be widened from the village level to include the neighbouring hilly areas; including only these people in the denominator would reflect a more realistic API calculation, as present calculations based upon administrative boundaries greatly deflate the API value in endemic areas.

### Primary health centres and villages with high case incidence

**Fig. 5:**
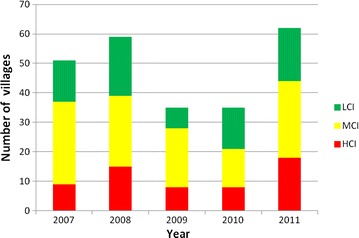
Number of villages with high case incidence (*HCI*), middle case incidence (*MCI*) and low case incidence (*LCI*) in the period 2007–2011 in Purworejo District.

**Fig. 6:**
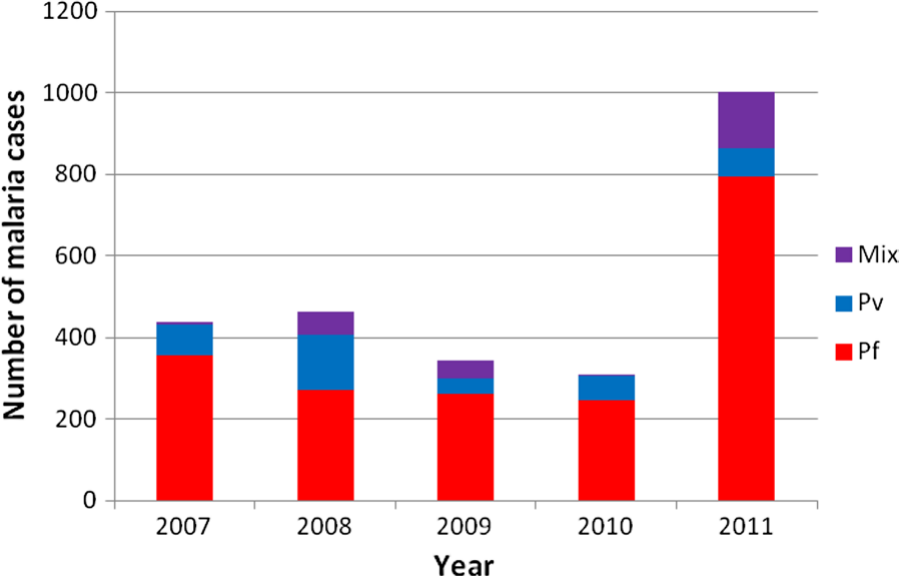
Number of malaria cases by species in 2007–2011. Pf = *Plasmodium falciparum*, Pv = *Plasmodium vivax* and Mix = mixed infection of *Plasmodium falciparum* and *Plasmodium vivax* (*Source: DHO, Purworejo 2007*–*2011*).

In Purworejo, a PHC may serve about 25 villages. Only one PHC was categorized as HCI from 2007 to 2010 and three PHCs in 2011 (Fig. 5). Data collected and recapitulated included cases by month, village, number of people with clinical malaria, age, sex, pregnancy status, *Plasmodium* species, ACT or non-ACT treatment, and indigenous or imported malaria cases. The proportion of malaria cases caused by *P. falciparum* was higher than *P. vivax* or mixed infections, that is 358/73/7, 273/135/54, 262/39/42, 246/61/2 and 795/69/138 in 2007, 2008, 2009, 2010, and 2011, respectively (Fig. 6). Malaria cases predominantly occurred in adults (more than 90%) (Table 3).

**Table 3:** The distribution of malaria cases in Purworejo District during 2007–2011 based on patient age

Age (years)	Total malaria cases in 5 years	Average annual cases	Proportion (%)
0–1	27	5.4	1.66
>1–4	256	51.2	15.72
5–14	617	123.4	37.88
15–64	1,418	283.6	87.05
>65	160	32	9.82
Total	1,955	325.8	100

*Source* DHO, Purworejo (January 2007–December 2011).

Indigenously acquired cases were higher than imported malaria cases. The proportion of men to women who had malaria was around 93–97% from year to year. Imported cases confirmed by epidemiological investigation—an investigation into the source of these imported cases that confirmed that they were all non-Java in origin—confirmed 35 of a total 132 cases during the 2007–2011 period (Source: DHO, Purworejo, 2007–2011). The low number of imported cases reflects the sub-optimal surveillance system. This was also supported by the fact that in 2009 and 2010 there were no data on active case monitoring as no VMWs was hired. Since 2007, malaria outbreaks occurred in more than ten locations, including the following villages: Tridadi (Banyuasin sub-district) in 2007, Kalitapas, Bleber (Bener sub-district) and Kembaran (Banyuasin sub-district) in 2008, Ketosari (Bener sub-district) and Ngrimun (Banyuasin sub-district) in 2009, Sudorogo (Kaligesing sub-district), Kalikalong (Loano sub-district), and Kedungpomahan (Kemiri sub-district) in 2010. As well as its adjacent areas, i.e., Kebumen, Wonosobo, Magelang, and Kulon Progo, the resurgence of imported cases usually occurred in specific patterns, such as in the middle and end of year, which is school holiday period in Indonesia, and also around the fasting month and the Idul Fitri, the biggest Moslem holiday in Indonesia. Here, family members who worked as migrant workers or transmigrants outside Java visit home temporarily. As most areas outside of Java are malaria endemic, the return of these people to their villages might spark malaria outbreaks as they might carry *Plasmodium* gametocytes. Malaria surveillance has been conducted in Purworejo to include monitoring human movement, although this was not supported by sufficient human resources because of lack of financial resources.

### Health care system

Purworejo had 27 PHCs, 64 auxiliary PHCs, one referral hospital, eight private hospitals and clinics, and around 307 midwives. Approximately 120 village midwives are placed in malaria endemic sub-districts. In 2010, 20 of 27 PHCs were reported free of malaria. Structurally, PHCs, auxiliary PHCs, and village midwives were working under the supervision and coordination of the DHO. The district referral hospital, in terms of management, was not under DHO, but local district government. For the purpose of disease surveillance, it is required to report routine morbidity cases (including malaria) to the DHO. To support the clinical treatment of malaria at the hospital, DHO allocates anti-malaria drugs to the district hospital.

### Malaria diagnosis

Malaria diagnosis was confirmed microscopically by trained microscopists from the DHO. A hierarchical system aims to attain correct diagnosis treatment, e.g., a village midwife would ask PHC’s microscopic personnel for a malaria slide diagnosis, and the PHC microscopic personnel could cross-check the slides with the ones in the DHO. Blood slide reading proficiency tests were performed regularly by re-reading positive malaria slides and 10% of negative malaria slides from the PHCs in the DHO laboratory. Similarly, DHO slides were cross-checked in the Province Health laboratory. However, routine malaria slide cross-checks could not be performed regularly because there was no allocated budget for sending the slides to Province level. The referral hospital, private hospitals and clinics did their own malaria identification based on microscopic or RDT in their own laboratories. Although sending slides to the DHO for cross-validation is not an obligation, cross-checking of malaria blood films should be a concern in confirming malaria diagnosis and case management. Inappropriate diagnosis would lead either to over- or under-diagnosis and subsequently over- or underestimate malaria status in some areas.

Malaria slides were usually prepared in the PHCs, referral hospital or in DHO laboratory as thick blood smears only; thin smears were typically not prepared. Because of this, species may be misidentified by inexperienced microscopists, which affects treatment protocol. *Plasmodium* species identification would affect treatment particularly in the administration of primaquine when co-administered with ACT. Village midwives or VMWs were also able to do finger prick for malaria smears, but slide staining is conducted by the PHC/referral hospital/DHO laboratories. Unfortunately, slides from village midwives or malaria workers sometimes took more than 3 days to reach the PHC laboratory for processing as it depended on the availability of transportation or on the mobility of the midwives or VMWs to the PHCs or laboratories, resulting in delayed diagnosis and treatment and thereby increasing the probability of ongoing transmission of parasites. Besides passive case detection conducted by PHC health workers, active case detection was done by VMWs. Malaria was suspected when individuals showed clinical symptoms such as fever, shivering, sweating, headache, and/or convulsions. Blood examination was performed once (with rare repeated blood slide examination). Some health workers included examination of individuals who complained of diarrhoea, nausea, vomiting, feeling weak, and those that had recently returned from malaria-endemic areas. Health workers in malaria-endemic villages often repeated finger prick blood examinations the following day to validate initial results. However, those who came from non-endemic areas or whose areas had been free from malaria for a few years did not perform this practice.

### Artemisinin-based combination therapy

ACT (artemether and lumefantrine or Coartem^®^) was introduced in Purworejo in 2004. When the study began in 2010, besides ACT, other malaria drugs being used included chloroquine or sulfadoxine and pyrimethamine (Fansidar^®^) in combination with primaquine. ACT procurement was managed by the malaria programme manager of the DHO, but chloroquine, Fansidar and primaquine were procured by pharmaceutical warehouses (*Gudang/InstalasiFarmasi*). PHCs were able to procure ACT directly from the malaria programme manager. ACT was only available at the DHO, and as a consequence, the PHC would contact the DHO only when ACT was needed. District pharmaceutical warehouses did not store ACT or perform ACT procurement and pharmacies were not allowed to sell ACT drugs. When this study began, it was observed that not all medical doctors and other health personnel at referral or private hospitals and clinics were aware that ACT was the first-line malaria drug in the national malaria control programme. Therefore, the demand for ACT was dependent on whether or not the clinicians in the hospitals/clinics had updated information related to ACT. Only clinicians that also served in PHCs in malaria-endemic areas used ACT to treat malaria infections.

ACT drugs came into two treatment regimens, i.e., artesunate-amodiaquine (AAQ) and dihydroartemisin-piperaquine (DHP) in combination with primaquine (PMQ). According to the national guidelines, to treat *P. falciparum*, amodiaquine was administered at 10 mg/kg body weight, artesunate was 4 mg/kg body weight and the PMQ was given on the first day with 0.75 mg/kg body weight for gametocyte clearance; to treat *P. vivax,* the same administration was given except that the PMQ was given from day 1 to 14 in 0.25 mg/kg body weight [25]. During the study observation period, not all health workers in PHCs, hospitals and clinics were familiar with this treatment regimen. Those who were familiar with ACT administered it either with or without PMQ. There was also uncertainty whether ACT should be prescribed to *P. falciparum* only, or also be used to treat *P. vivax* because of uncertainty regarding the resistance status of *P. vivax*. Health workers and VMWs from endemic areas were more familiar with ACT compared to those from non-endemic-malaria villages. Re-education, literature dissemination and standard operating procedures in malaria treatment are needed.

A study to evaluate the efficacy and side effects of AAQ + PMQ and DHP + PMQ treatment in uncomplicated *P. falciparum* malaria in Purworejo [26] found no early or late treatment failure of ACT. It is also interesting to note that both regimens had no differences when looking at the elimination of the sexual forms of the parasites (gametocytes). Although all parasites were killed on day 3, gametocytes remained present in subjects treated with either AAQ + PMQ or DHP + PMQ during the regimen (days 1–2). Therefore, protection of patients from mosquito bites during medication (days 1–3) might be useful to prevent transmission.

### Other malaria control activities in Purworejo

From 2007 to 2011, Purworejo applied selective IRS, bed net distribution, bed net re-impregnation activities (in 2011 only), and LLIN distribution, active case detection by VMWs, and mass blood/fever surveys (Table 4). IRS was conducted based on geographic factors and presence of confirmed malaria vectors whereas bed nets were distributed following priorities, i.e., for pregnant women and their babies in malaria outbreak areas, gametocyte carriers, and for all households in a village during outbreaks. However, it is difficult to measure whether these interventions had a significant impact on malaria transmission due to lack of documentation.

**Table 4:** Comparison of interventions towards malaria in Purworejo and Wonosobo District Health Offices during 2007–2011

	Purworejo					Wonosobo				
Year	Cases	IRS	LLIN	VMW	MBS/FS	Cases	IRS	IBN	VMW	MBS/FS
2007	408	12,000	3,000	50^a^	120/120	133	25,000	1,300	50	0/16
2008	439	12,000	2,000	50^a^	120/120	160	25,000	3,000	38	0/16
2009	349	8,400	1,000	30^b^	60/60	80	25,000	3,000	33	0/16
2010	353	6,400	800	30^c^	35/30	129	25,000	700	33	0/16
2011	1,001	4,600	800	30^c^	30/9	84	25,000	0	32	0/16

*Source* DHO, Purworejo, 2011.

*IRS* houses sprayed with indoor residual spraying, *LLIN* number of distributed long-lasting insecticidal nets, *VMW* number of Village Malaria Workers, *MBS* Mass Blood Survey, *MFS* Mass Fever Survey, *IBN* number of impregnated bed nets.

^a^10 months.

^b^ 8 months.

^c^ 6 months period of work annually.

A village health forum (*Forum Kesehatan Desa*) and Vigilant Village Programme (*Desa Siaga*) supported by the Ministry of Health were neglected as malaria cases decreased relative to when the forum was formed in 2004. Regulations that support the elimination of malaria in the district were promulgated in 2010. This regulation encouraged each village to develop a team for malaria surveillance related to human migration. However, only a few villages successfully developed and implemented activities based upon this regulation. Thus, few reports on human migration were reported in most villages. As API in Purworejo started to increase from 2005 to 2006 (from 0.42 to 0.55 per 1,000) (Source: DHO, Purworejo), cross-sector networking between the health departments and the Department of Transportation at district level was initiated. These activities aimed to oversee the migration of people through bus agencies. The bus agencies in Purworejo were expected to report the number of people moving in or out of the district, particularly movement to and from islands outside of Java. Buses have advertisements informing people of malaria, where testing can be done and where malaria is endemic. However, effectiveness of this initiative was questioned due to limited documentation and reports.

As a part of Gebrak Malaria activity, DHO of Purworejo, together with neighbouring districts initiated cross-border activities. The main purpose was notification of the increase of malaria cases within each territory and included informing the neighbouring district’s PHCs when people sought malaria treatment or when there were instances of malaria-related hospitalization in their territory. This networking facilitated follow-up of patients when they returned to their villages.

### Limited financial sources of malaria programme at district level and strategies to anticipate, a lesson learnt from Wonosobo, the adjacent district

Similar to other vertical disease control programmes in the Ministry of Health, malaria control programme activities at district level are supported by three main government sources, namely the central, province and local (district) funds. Each funds has different allocation. The district budgets were used to support drug procurement, bed nets, operational IRS, re-treatment of ITNs, VMWs, Mass Blood Survey and Mass Fever Survey. Some activities had overlapping funding with provincial budget, such as IRS, bed net procurement or purchase of malaria drugs. In the case of procurement of malaria drugs, no clear division of tasks was set causing risk of stock-outs or drug expiry.

Since 2000, Indonesia has applied a decentralized governance system in most sectors, with the result that each of Indonesia’s over 500 districts is responsible for prioritizing and carrying out health activities. In consequence, the greatest percentage of funding is from district government funding (*APBD II*), followed by provincial government funding (*APBD I*) and then from general and specific purpose funding (*Dana Alokasi Umum*, *Dana Alokasi Khusus*). Health budgets, including malaria, are decided by local/district government (*APBD II*) (Table 5). However, the district health budget *APBD II* does not always reflect the local requirements and diseases [27]. In 2003, MOH has released regulation to strengthen surveillance at the district level which unfortunately was uneasy to be implemented [28]

**Table 5:** Malaria funding in US$ provided by local government (District level) through APBD II in Purworejo and its adjacent areas compared to their annual parasite incidence (cases per mil) from 2007 to 2011

Year	Purworejo		Wonosobo		Magelang		Kulon Progo		Kebumen	
	APBDII	API	APBDII	API	APBDII	API	APBDII	API	APBDII	API
2007	40,391	0.57	14,669	0.16	NA	NA	NA	0.21	3,422	3.82
2008	29,339	0.61	4,889	0.21	6,552	0.03	45.476	0.16	2,640	0.21
2009	10,366	0.47	9,584	0.02	12,322	0.02	32.078	0.29	1,466	0.1
2010	5,281	0.49	977	0.16	19,462	0.18	25.819	0.06	733	0
2011	5,867	1.31	977	0.01	20,537	0.012	6,454	0.32	1,466	0

*Sources* DHO Purworejo, Wonosobo, Kebumen, Magelang and Kulon Progo (1US$ was approximately 10,225 rupiahs (30 June 2009).

*APBD* Anggaran Pembangunan Belanja Daerah, *API* annual parasite incidence, *NA* non available data.

Coincidently, a few years before 2000, malaria, which has been almost disappeared after Malaria Eradication Operation Command (*Komando Operasi Pembasmian Malaria* or KOPEM)—set up in 1962 and resumed in 1972 [29]—began to rise in Menoreh area. Purworejo, Kulon Progo and Kebumen received *Inisiatif Anti*-*Malaria Indonesia* (IAMI) relief [30], but not Wonosobo, as malaria cases was considered to be low. This situation gave an advantage in the future, as Wonosobo used to be independent particularly in develop strategies to gain potential funding in financing their malaria programme activities. Budget constraints spurred Wonosobo to prioritize early case detection as the most important action to prevent escalation of malaria cases. As a consequence, sufficient number of VMWs was need to be maintained.

Compared to Purworejo that lost almost half of its VMWs in 2009, Wonosobo was able to maintain approximately the same number (Table 5). Although both districts faced budget cuts, they used different strategies to try to maintain staff, with Purworejo choosing to reduce the working period from 12 months to 8–10 months per year in 2010 and 2011, while Wonosobo maintained staff numbers by simply cutting salaries (Source: malaria programme officer of DHO, Wonosobo, pers. comm.). Therefore, Wonosobo DHO responded to the increase of malaria by retaining a relatively large number of JMDs using available, modest, regional budget.

Compared to Purworejo, budget reductions in Wonosobo were greater (see Table 4). The malaria budget from *APBD II* in Purworejo declined by half from 2009 to 2010 (from US$10, 366 to 5,281; US$1 = approx. 10,225 rupiahs in 2009) primarily due to the reduction in malaria cases from 2007 to 2009. Meanwhile malaria cases in Wonosobo fluctuated from 2007 to 2011, with cases decreasing by half in 2009 (from 160 cases to 80 cases). Despite this success (or because of it), district government funding (APBD II) was cut sharply to one-tenth of the previous year.

Besides reduced VMW salaries as explained previously. Wonosobo also used the previous year’s residual budget to distributed ITNs and carry out IRS. Other funds came from province were used for ITN procurement. Additional funds for IRS were obtained from the Directorate General of Disease Control in the MOH in response to a proposal to contain malaria outbreaks. Wonosobo also used its Health Operational Fund (*Bantuan Operasional Kesehatan* or BOK) budget for malaria control; this fund was provided by central government through the MOH for prevention programmes at PHC. Although many DHOs and PHCs interpreted the regulations for BOK funds as being restricted to Maternal and Child Health programmes, Wonosobo interpreted the regulations as allowing use of the money for activities, such as: (1) diagnosis and case management; (2) spot surveys of vector breeding places; (3) vector control; and, (4) distribution of bed nets to populations at risk. Wonosobo, in addition, assisted by the Vector Research Centre of Salatiga and Banjarnegara, performed a study on mosquito bionomics and supported vector surveillance stations in Kepil, Kalibawang, Wadas Lintang, Kaliworo and Sidoharjo villages for several years. The results of this surveillance were used as a guide for implementation of vector control in the district, to confine the local government and cross-sectoral authority (department of Public Work, a non-health sector) in procurement of the funding and the provision of facilities, such as: (1) distribution of *Apocheiluspanchax* fish in paddy fields and a shift from rain-fed irrigation systems (*sawah tadah hujan*) to irrigation systems aimed to reduce populations of *An. aconitus*; and, (2) based on evidence that most mosquito vectors in Wonosobo (*An. aconitus*, *An. maculatus* and *An. balabacencis*) were biting outdoors after dusk, public restrooms and bathing rooms that reduced mosquito exposure while bathing after dusk were built (Source: DHO, Wonosobo, Junaidi, pers.comm). Decline of malaria cases in Wonosobo during 2007–2010 seems supported by the capacity to formulate a policy, out of the box strategies in seeking potential financial resources and relying on evidence based findings to do vector control measures.

### Constraints and challenges

**Fig. 7:**
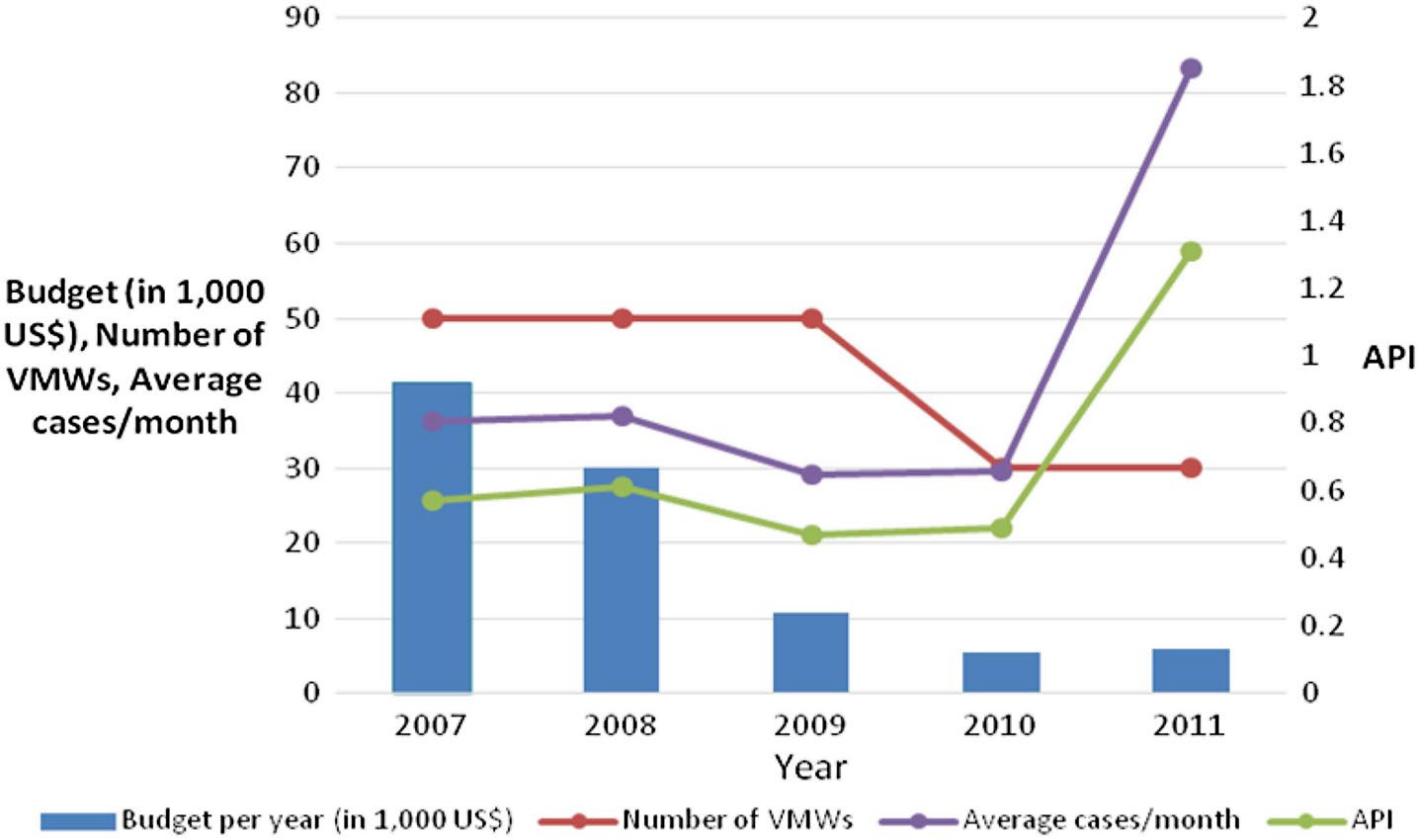
Inverse relationship between available resources and malaria endemicity in Purworejo District (2007–2011). Decreased district budget for malaria and number of VMWs (village malaria workers) were followed by the increasing malaria cases/month and API (annual parasite incidence) in 2010.

The Wonosobo malaria control programme operates in a complex decentralized environment. While technically autonomous, the success of the programme depends upon cooperation with other sectors and advocacy to and negotiation with both executive and legislative parts of district government. Such advocacy is continuous: i.e., members of the health commission in the local parliament might change every 2 years, requiring renewed advocacy with every membership rotation to ensure that malaria elimination is supported. Along with Wonosobo, Magelang and Kulon Progo districts were also successful in getting funding for their malaria control programmes. Even though malaria cases decreased over the last 5 years, the DHOs of Magelang and Kulon Progo’s malaria funding was sustained at levels to allow hiring of malaria workers and village cadres/volunteers for surveillance activities. In contrast to Purworejo, VMWs were continuously on duty in Wonosobo, Magelang and Kulon Progo. The decline in malaria cases in Purworejo led to reduced funding, fewer resources for VMWs, and a small absolute increase in the API from 0.49 to 1.31 (Fig. 7). This is a conundrum faced by any elimination strategy: the only cost-effective exit strategy is success, otherwise the programme will be in a cycle of decline in cases, followed by a decline in commitment and funding, then resurgence.

## Discussion

The formal MOH target for malaria elimination from the island of Java is the end of 2015 [31]. Although the programme has made good progress, it seems unlikely that this target will be achieved for Kulon Progo and Purworejo districts, in particular. This study has described a malaria control programme at district level from multiple perspectives: technical, financial, political, and social [32, 33].

There is little doubt that malaria control has succeeded in Purworejo, with a dramatic decline in passively reported incidence from an API of 35 in 2000 [21] to an API of 0.49 in 2010 (Source: DHO, Purworejo). These figures may underestimate actual malaria incidence because they do not include asymptomatic infections or those who seek treatment outside the public health system. Population [34] and school-based cohort studies from 18 primary schools [35] in Purworejo (December 2008 to June 2009) reported considerably higher incidence, although the population was selected from a focus of known malaria transmission. Whatever the absolute level of transmission, Purworejo has not yet been able to reduce transmission from very low levels to zero during the past several years.

While multiple factors contribute to the failure (so far) to achieve elimination in central Java and Purworejo, one important obstacle is the difficulty of coordinating malaria control activities across administrative boundaries. Endemic areas of the Menoreh hills have similar geomorphology and vector dynamics but this area incorporates a number of districts, each of which has enjoys administrative autonomy. While autonomy can foster innovation and increase motivation, it can inhibit coordination needed to attack malaria in areas with similar epidemiology. To improve effectiveness of interventions, Purworejo could share mapping, entomological and epidemiological data and intervention strategies with adjacent districts. For instance, stratification of incidence by village might reveal a pocket area near borders, where adjacent villages in different districts might have similarly high levels of transmission. Similarly, information on vectors found in foci of transmission could be shared, including information on what interventions are effective or not. Micro-ecology of vectors even in nearby villages needs to be recognized and managed. Interaction of micro-ecology and natural or artificial ecosystem might result in adaptation of Anopheles breeding sites. As different areas might vary in both factors, thus understanding the vectors in term of their abundance, seasonal distribution, biting, resting and breeding behaviours is important for effectiveness of vector control measures [36]. While it is an administrative requirement that each district fund and formulate its own control strategy, the task would be made easier and more effective if adjacent districts with similar malaria epidemiology were to share data, experience and plans. Affected districts might work together informally, or it may be that the Provincial Health Office of Central Java might assist in leading the process. Detailed documentation and reporting related to entomological and epidemiological data, and intervention that ever done either in district or inter-district level would be valuable to evaluate whether a strategy works well or vice versa.

Funding sources in Indonesia are many, with capitation funds, health centre operational funds, and district, provincial and national funds all available, but via different mechanisms for use by local governments. Collaboration might improve the chances of leveraging sufficient funds for malaria elimination.

### Malaria diagnosis and reactive case detection

From a technical standpoint, the most basic component of a malaria elimination programme is accurate information on the distribution of malaria cases. Purworejo has taken steps to ensure that diagnosis is accurate via establishment of a malaria cross-check (QA) system. As cases become rarer, it becomes important to ensure microscopists and surveillance staff increase their motivation to follow up and treat individual cases. To this end, an elimination mentality must be established in communities and in health centre staff. Surveillance must become more intense as cases diminish, which is counter to normal public health practice. Further, the district government will need to ensure that hospital-based cases and those treated by private practitioners are followed up. Purworejo has not yet instituted reactive case detection, whereby family members and neighbours of cases are screened and treated for malaria. It may be that such efforts are not needed, but it should be noted that reactive case detection is a key part of the elimination programme in Sabang, Aceh, Sumatra. Case investigation to determine the locus of transmission is also essential as this will determine where to focus control effects within district transmission or which populations might be targeted for migration surveillance for transmission occurring outside the district. In Wonosobo, migration surveillance was effectively carried out by VMWs, as the villagers inevitably knew who had travelled to which area and when they returned home. This activity could be replicated at other districts by also involving local neighborhood to document and report people mobility to anticipate a new transmission.

### Vector control

The vector species of the Menoreh Hills may be largely exophilic, but they are also not especially efficient vectors of malaria. Purworejo has focused its LLIN distribution in foci of transmission to reduce the probability of transmission. Unfortunately, its vectors, with the exception of *An. aconitus*, are not especially vulnerable to larval control. Nonetheless, in certain foci of transmission, it may make sense to reduce transmission by focused larviciding or environmental management.

### Sub-patent parasitaemia

Detection of sub-patent parasitaemia may or may not be essential for malaria elimination, depending upon the probability of onward transmission by low-density parasitaemias. That such transmission can occur is not in doubt, however, the key question is how often it occurs and what can be done to minimize it short of detecting the parasite and killing it with drugs. Clearly, vector control efforts are key to minimizing onward transmission, as described above. However, newer diagnostics such as extra-sensitive RDTs [37] or LAMP [Loop-mediated isothermal amplification] assays may become available to allow direct detection of low density parasitaemias, should this be necessary.

## Conclusion

At the end of the study period, Purworejo was still in pre-elimination stage as API had remained low but undiminished in recent years. Weaknesses in surveillance and focused case investigation are likely a root cause of the lack of progress; this is related to lack of political commitment by the local government, despite the enthusiasm and competence of the DHO. Based on this study, several strategies are proposed that might be adopted for achieving the goal of malaria elimination in Purworejo. These include: (1) redefining foci of malaria, i.e., using ‘total population living in the Menoreh hills area’ as the denominator instead of ‘total district population’ when calculating the API; (2) encouraging collaboration between district governments to innovatively use potential funding to support malaria surveillance; (3) revising the decentralization policy by strengthening the role of the province to improve coordination across districts effectively; (4) developing good case investigation and reactive case detection systems; and, (5) strengthening the involvement of the private sector for malaria surveillance, particularly in case reporting.
